# Psychological first aid for Ukrainian civilians: protocol and reflections on a volunteer international phone-based intervention

**DOI:** 10.3389/fdgth.2025.1539189

**Published:** 2025-04-14

**Authors:** Yanina Shraga, Helen Pushkarskaya, Orly Sarid

**Affiliations:** ^1^ The Spitzer Department of Social Work, Faculty of Humanities and Social Sciences, Ben-Gurion University of the Negev, Beer-Sheva, Israel; ^2^ Department of Psychiatry, Yale Medical School, New Haven, CT, United States

**Keywords:** informal international organizations, Psychological First Aid (PFA), phone-based therapeutic interventions, protocol & guidelines, realist autoethnographic reflective report

## Abstract

Informal mental healthcare groups often provide telephonic and text-based interventions to support communities affected by natural and man-made disasters. Operating outside formal regulations, these groups offer flexible and innovative care; documenting their practices is crucial for evaluating service quality. This paper presents a protocol of an international, informal Psychological First Aid (PFA) telephone-based initiative and a reflective account from a volunteering therapist. The initiative aimed to support Ukrainian civilians affected by the Russian-Ukrainian war through crisis intervention and resilience-building. Guided by PFA principles, theoretical first aid models, and evidence-based practices, the telephone interventions focused on reducing distress, providing moral support, and restoring functioning. A Russian-speaking therapist assisted 34 Ukrainian civilians, primarily addressing acute stress, anxiety, and panic attacks. Using an autoethnographic approach, this study integrates the therapist's retrospective reflections, cultural context, and professional learning to examine PFA implementation in a crisis setting. Individuals who received telephone-based PFA reported decreased distress and enhanced coping strategies, suggesting effectiveness. The initiative's three-year continuation illustrates its sustainability. The therapist's reflections highlight the importance of professional preparation, shared linguistic and cultural backgrounds, and a sense of purpose in delivering effective support. While limitations include the absence of standardized measures and potential self-selection and volunteer biases, this study demonstrates the feasibility of providing remote psychological first aid to civilians through informal international groups. Critically evaluating practices adopted by these informal organizations is essential for understanding their effectiveness, improving future implementation, and co-creating best practices for crisis intervention and support services that embrace “Care Without Address” models.

## 1 Introduction

Psychological First Aid (PFA) is an evidence-based approach endorsed by the World Health Organization (WHO) to provide immediate emotional and practical support to individuals experiencing crisis-related distress, including war and displacement (1). Unlike formal psychotherapy, PFA prioritizes stabilization, safety, and connection, helping affected individuals feel supported and guided toward appropriate resources (2). The WHO's Look, Listen, Link framework emphasizes recognizing distress signals, offering compassionate listening, and connecting individuals to further care (1). PFA is particularly critical in war-affected populations, where individuals endure acute trauma, uncertainty, and loss (3). By equipping volunteers and frontline responders with essential crisis intervention skills, PFA plays a crucial role in mitigating immediate distress and fostering resilience, laying the foundation for long-term recovery (4).

The rise of telehealth has revolutionized mental healthcare delivery, enabling innovative, scalable, and accessible interventions, including those based on PFA. This transformation accelerated during the COVID-19 pandemic, shifting traditional, location-based care models toward “Care Without Address” approaches, which prioritize accessibility over physical infrastructure (5). Telehealth has proven particularly valuable in crisis settings where formal mental health services are inaccessible, unaffordable, or unreliable (6). Informal volunteer-based PFA programs operating through digital platforms have pioneered highly adaptable and scalable approaches to emergency psychological support. Documenting and critically evaluating practices adopted by these informal organizations is essential for understanding their effectiveness, improving future implementation, and co-creating best practices for crisis intervention and support services that embrace “Care Without Address” models.

On February 24, 2022, the Russian invasion of Ukraine triggered widespread psychological distress, exacerbating existing mental health vulnerabilities. Ukrainian civilians faced resource shortages, displacement, and profound uncertainty, with many women and children fleeing to western Ukraine and neighboring countries such as Moldova, Poland, and Slovakia (7). Although Ukrainian mental health professionals had prior training in cultural and clinical trauma, addressing the unique complexities of war-related trauma—including displacement, combat exposure, loss, and atrocity—required additional expertise (8). To address longstanding regional needs, especially in Donetsk and Luhansk, the Ukrainian government approved a national mental health reform plan in December 2021 (8). However, just months later, the escalation of conflict damaged healthcare infrastructure, displaced professionals, and further depleted the already limited pool of trauma specialists, creating a critical gap in mental health care (7).

**Figure 1:**
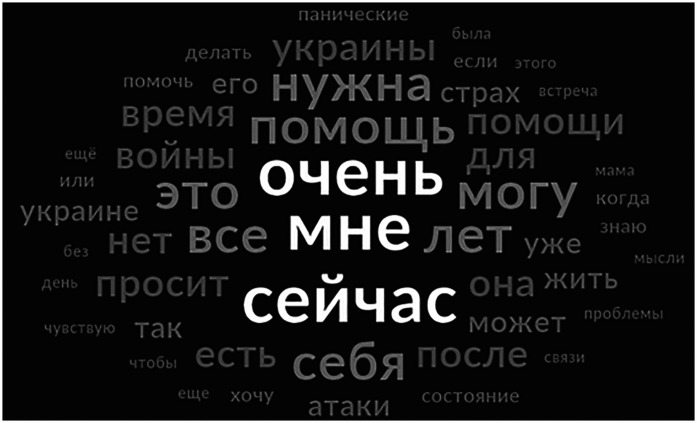
Word cloud of 50 most used words (in Russian) in callers’ requests received by IRIS between February and December 2022. Most used words: “***очень мне сейчас*** *нужна помощь*” = “***really*** ***right now I** need help*”. Total 2,505 requests were received using a digital intake form by IRIS coordinators between March 1st and December 31st, 2022. The form included a question “What help do you need?” Answers to this question were analyzed using NVivo 1.7.1. Figure depicts a resulting word cloud of 50 most used words.

In response, on February 24, 2022, an Israeli social worker mobilized Russian-speaking mental health professionals to provide telephone-based PFA to Ukrainian civilians. Initially, 15 professionals shared their personal phone numbers on Facebook, offering free psychological support via WhatsApp, Viber, and Telegram. This initiative, Increasing Resilience Israeli Support (IRIS), within six weeks expanded to 200 volunteers and has continued operating for the past three years, demonstrating sustainability. In February 2022, more than 50 individuals were contacting IRIS daily for assistance. Between March 1st and December 31st, 2022, a total of 2,505 calls were received. Figure 1 depicts 50 most used words in these requests, illustrating urgency of their needs.

The purpose of this paper is to leverage experience of an IRIS volunteer to contribute to the development of best practices for crisis intervention and support services that transcend geographic barriers by:

(1) Presenting the protocol of an international informal telephone-based PFA initiative.
(2) Using a realist autoethnographic approach to reflect on its real-world implementation through a volunteer's experience.

Throughout this paper, we use the following terms: “therapists” for all volunteers, irrespective of their formal qualifications, “initiative” for the informal international telehealth-based PFA initiative IRIS, and “callers” for all individuals seeking assistance. All procedures were developed in accordance with recognized PFA guidelines (1, 9) and were approved by the Institutional Ethics Committee of Ben-Gurion University of the Negev.

## 2 Methods

First, we outline IRIS's objectives, organizational structure, operational procedures, therapeutic approach, and the strategies used to implement this approach through digital platforms. Next, we adopt a realist autoethnographic approach (10–13) to examine the implementation of Psychological First Aid (PFA) in a crisis setting, based on the reflections of a volunteer. This will include a narrative case study of one of the individuals who receive PFA through the initiative, providing a concrete example of how therapeutic strategies were applied and experienced in practice.

*Autoethnography* combines first-person narrative with cultural analysis (14, 15), aiming to provide an insider's factual account of an organization, a social group, or cultural practice while minimizing the researcher's subjective influence. A *realist* approach to organizational *ethnography* emphasizes objectivity and factual accuracy (12), making it well-suited for evaluating IRIS's operational procedures and therapeutic strategies to inform future implementation of crisis interventions aligned with “Care Without Address” models of social services. The volunteer's long-term engagement provides valuable empirical insights into operational procedures and social practices that are hidden from public view (16). The therapist's reflections on therapeutic models, presented through a narrative case study, offer a deeper understanding of the process of therapy (17). These observations are integrated with a cultural analysis of how the therapist's linguistic and cultural background shaped therapeutic interactions, how shared Soviet and post-Soviet norms facilitated rapport-building, and how prior professional experiences informed the delivery of PFA to war-affected populations.

A realist autoethnographic approach can provide critical insights when more quantitative methods, such as large-scale surveys of volunteers or the administration of standardized distress-assessment instruments before and after an intervention, are not feasible for practical or ethical reasons. Quantitative methods are often limited by logistical constraints, including sample size, access to participants, and the sensitive nature of the context in which crisis interventions occur (11, 18). For instance, in crisis settings, individuals seeking assistance may be experiencing acute psychological distress, making it ethically problematic to subject them to standardized assessments that could exacerbate their symptoms or compromise their willingness to engage with support services (19). Thus, in contexts where quantitative methods are limited by feasibility or ethical concerns, realist autoethnography offers a powerful tool for generating actionable knowledge and informing best practices in crisis intervention.

*A narrative case study* allows for a detailed exploration of the therapist's subjective experience of interacting with a caller, highlighting how the intervention was conducted, how the therapeutic relationship evolved over time, and how the caller responded to the intervention (20, 21). This approach captures the nuanced emotional and relational dynamics involved in PFA, providing deeper insight into the effectiveness and emotional impact of the intervention (22, 23). Additionally, it helps identify adaptive strategies and challenges encountered during the intervention, offering practical guidance for improving future crisis responses (24, 25). Narrative case studies are particularly effective for examining complex, context-dependent phenomena where human relationships and emotional responses play a central role (22, 23).

## 3 Protocol for the informal international telephone-based PFA initiative

### 3.1 Objectives, organizational principles, and therapeutic approach

#### 3.1.1 Objectives

The initiative focused on addressing mental distress and promoting resilience among civilians affected by war. War represents an extreme ordeal that impacts entire populations, causing severe mental distress, including anxiety, depression, fear, anger, and hopelessness among civilians in conflict zones (26). However, individuals coping with the aftermath of war can also demonstrate resilience, with hopefulness being a critical predictor of recovery (27). To foster resilience, therapists prioritized nurturing hope and conveying a steadfast commitment to ongoing support.

#### 3.1.2 Organizational principles

Volunteers were professionals in psychology, psychiatry, social work, or art therapy. Participation was voluntary. Psychological first aid typically consisted of 1–4 sessions per caller. Interventions followed the PFA guidelines aiming to reduce distress, provide moral support, and restore functioning. For cases requiring extended care, callers were referred to local services.

#### 3.1.3 Psychological first aid model

The initiative's therapeutic approach aimed to alleviate mental distress and foster resilience by following Psychological First Aid (PFA) guidelines (1, 9), theoretical first aid models (3, 28–32), and evidence-based research. It relied on studies indicating that early cognitive-behavioral interventions can prevent PTSD and reduce depressive symptoms (2, 32, 33). Digital interventions, including phone- and internet-based approaches, have also demonstrated effectiveness (34–40). This section briefly summarizes the key PFA models that informed the initiative's protocol.

Hobfoll et al. (3) developed an evidence-based PFA framework, Five Principles, integrating research and expert consensus to establish five core principles for building resilience: (a) promoting a sense of safety; (b) calming the individual; (c) enhancing feelings of capability, self-efficacy, and familial and communal robustness; (d) strengthening beneficial social ties; and (e) creating hope. Hobfoll's COR theory (28), which centers on the preservation and restoration of existing resources to mitigate stress and enhance overall resilience. This model ensures that interventions prioritize conserving and rebuilding personal and environmental resources, thereby preventing resource loss, which can exacerbate stress (28).

Farchi et al. (29, 30) introduced the Six Cs Model—a framework for immediate cognitive psychological first aid —imed at helping an affected individual transition from a state of helplessness and passivity (i.e., the “freeze” response) to a functional and active state shortly after a traumatic event. Six Cs stand for Cognitive Communication, Challenge, Control, Commitment and Continuity. While Hobfoll's COR approach emphasizes psychological stabilization and the conservation of resources, the activation–oriented SIX Cs model encourage individuals to be active and develop effective functioning. It prioritizes Cognitive Communication to diminish emotional overwhelm and bolster cognitive functioning. It introduces a Challenge for active engagement, Control for cognitive management of situations, Commitment from the helper to reduce loneliness, and Continuity to ensure narrative coherence.

Lahad et al. (31) developed the BASIC PH model, which highlights individual differences in preferred coping styles and emphasizes that resilience is strongest when multiple mechanisms are available. The BASIC PH model identifies six coping strategies: Belief (spirituality, meaning-making), Affect (emotional regulation), Social (relationships, support systems), Imagination (creative coping), Cognition (rational problem-solving), and Physiological (body-based strategies like breathing and movement). Lahad's model guides discussions with affected individuals about possible coping strategies and helps them recognize new ways to manage stress.

Ruzek et al. (32) developed the Core Actions model of PFA, which consists of eight key actions: contact and engagement, safety and comfort, stabilization, information gathering, practical assistance, connection with social support, coping support, and linkage to collaborative services. Ruzek's PFA provides a structured intervention focused on reducing immediate distress and promoting active adaptive functioning. Ruzek's model and the BASIC PH model formed the foundation of the initiative's hybrid approach, which combined initial stabilization (calming techniques) with effective coping strategies, ensuring a flexible, holistic intervention tailored to the evolving needs of callers.

### 3.2 Adaptation for a telehealth setting

The adaptation of PFA models for a virtual setting includes training on ethical concerns (e.g., confidentiality in digital spaces), managing technological barriers, and crisis escalation protocols to ensure safe and effective intervention.

#### 3.2.1 Ethical considerations

The initiative strictly followed ethical principles for the practice of telepsychology and PFA guidelines (1, 41). Confidentiality was ensured by using end-to-end encrypted platforms (WhatsApp, Telegram, Viber). The therapist-maintained session notes, text messages, and reflective journals were stored in password-protected files and restricted-access cloud systems; identifying details were removed.

Cultural considerations played a pivotal role in the initiative's approach, as they are crucial for the appropriate expression of empathy and the development of therapeutic rapport (42). Only Russian and Ukrainian speaking individuals were involved in the initiative. Volunteers with Russian phone numbers (recent immigrants to Israel or other countries) did not contact the callers directly, acknowledging the sensitivity of the situation. They either used their Telegram login or obtained a new non-Russian number. Calls were conducted respecting callers' language and gender preferences. The initiative included therapists experienced in addressing LGBTQ+ concerns. The initiative therapists, all immigrants from former USSR territories, shared with Ukrainian callers preserved over generations elements of Soviet cultural norms (43, 44). For instance, during the first conversations, callers exhibited considerable politeness towards the therapists, even in chaotic situations or while under fire. This politeness reflects ingrained in Soviet norms respect towards professional authority. Soviet culture dictates a restrained display of unpleasant emotions, such as anger or rage, fear and generally discourages expressing these emotions within families to protect loved ones (43). In contrast, anonymous conversations with therapists encouraged openness and candor. One male caller likened this therapeutic relationship to “*meeting a stranger on a train and talking to them about your troubles*,” a common phenomenon in Soviet culture.

#### 3.2.2 Technical considerations

Because of the technical limitations during wartime, the initiative prioritized telephone communication, considering it the primary means of contact (45). In case of call interruptions, incomplete sessions were rescheduled via texts. Free applications like WhatsApp, Telegram, or Viber were used to conduct calls and texts. Challenges included issues like poor internet or cellular service, the lack of technological literacy among some callers, and maintaining the pace of interventions (46).

#### 3.2.3 Crisis escalation protocols

All therapists were mental health professionals with training and experience in identifying escalating distress and in providing immediate emotional support and stabilization to callers, using active listening, maintaining calm communication, and ensuring a sense of safety. The initiative developed relationships and maintained communication channels with local agencies to facilitate follow-up or in person care.

### 3.3 Volunteer recruitment and training

All PFA volunteers were professionals in psychology, psychiatry, social work, or art therapy, primarily from Israel, but also from the U.S., and several European countries. Recruitment occurred through professional connections and social networks. The initiative's founder, a social worker, interviewed each volunteer to assess credentials and relevant experience. Volunteer participation fluctuated. While 15% (mostly therapists and social workers) remained consistently active throughout 2022, others participated intermittently or for shorter periods, with new recruits added as needed. Caseloads varied—in March 2022, the three busiest therapists handled 45, 30, and 28 calls, while most responded to fewer than 20. This pattern continued, with 15%–20% of therapists taking on the most active roles each month, rotating responsibilities over time.

The initiative prioritized therapists’ mental health and training needs, offering expert-led lectures and training. World-renowned trauma specialists conducted sessions 5–6 times a week in early 2022, later shifting to 1–2 times per month. These trainings followed the WHO-based PFA framework, emphasizing trauma-informed communication, cultural competence, and implementation using digital means. Various digital mental health interventions, including mobile apps and computer-based therapy, have proven effective in crisis settings (47). Successful approaches include clinical evaluation, psychoeducation, problem-solving therapy (PST), and cognitive-behavioral therapy (CBT), particularly for anxiety and depression (45). Such interventions also benefit war victims (47), often serving as the only practical means of delivering critical mental health support, including diaphragmatic breathing exercises and techniques for managing intrusive thoughts (18). All therapists received targeted training in these methods.

Clinical supervision and debriefing sessions were held 5–6 times per week via Zoom, covering case consultations, translation support, and resource-sharing. One organizer managed conflict prevention and resolution, fostering a supportive community and maintaining quality care.

### 3.4 Telephone-based implementation framework

**Figure 2:**
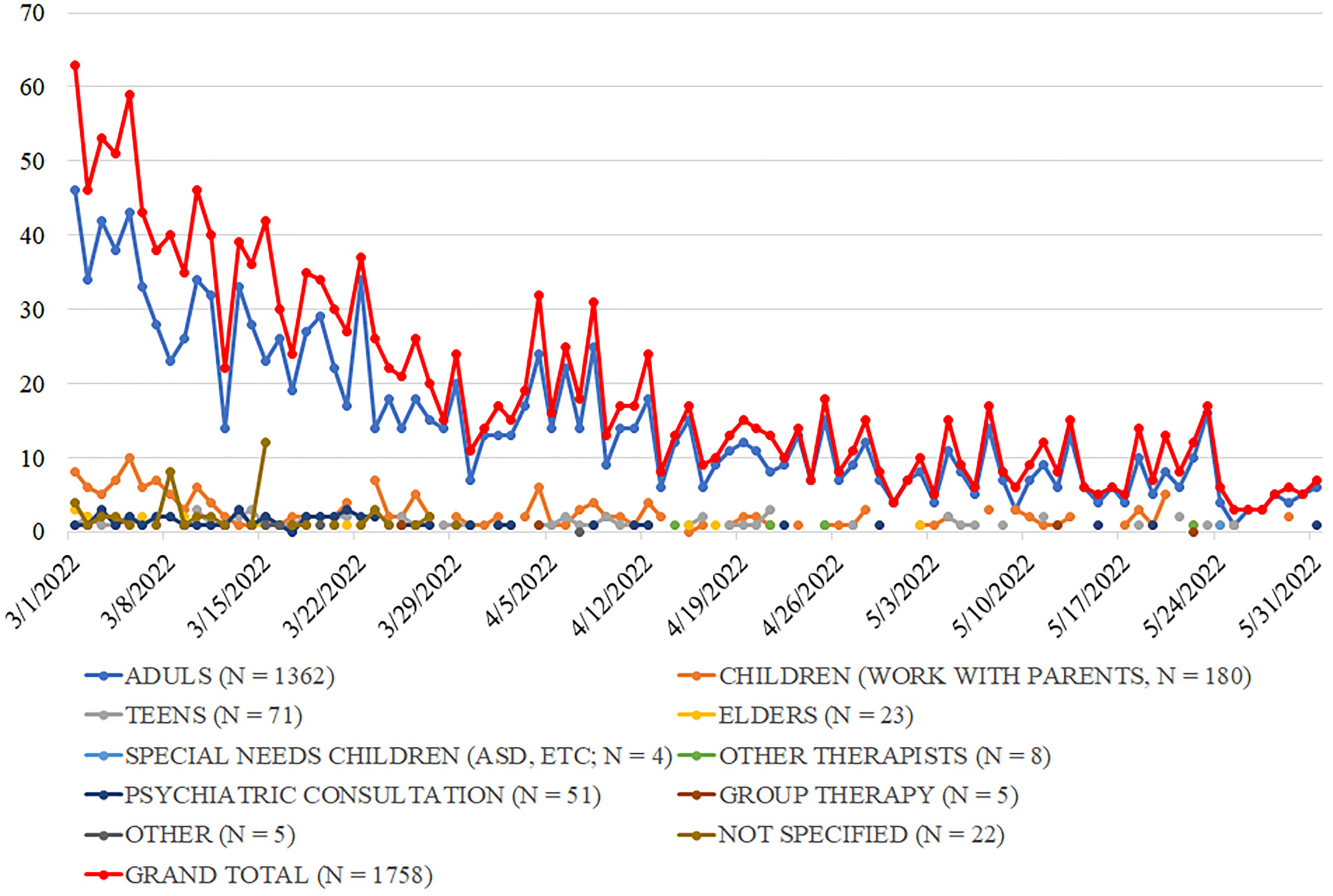
Call trends between march 1st and May 31st.

Only end-to-end encrypted platforms (WhatsApp, Telegram, Viber) were used for communication. In the first week, callers contacted therapists directly via personal phone numbers. To streamline operations, a digital request form replaced personal numbers on March 1. Call coordinators assigned numerical codes to requests, shared them with therapists, and therapists selected cases matching their expertise. Each call was assigned to a therapist. Complex cases were assigned to experienced trauma specialists. Coordinators tracked call statuses (“new,” “assigned,” “repeat,” “closed,” “no response,” “wrong number”) and therapist workloads. Of 2,505 calls received between March 1st and December 31st, 2022, 96 callers did not respond to the therapists' attempts to reach them, 13 provided incorrectly formatted phone numbers, and the remaining individuals spoke with a therapist. Therapists aimed to respond to each call on the same day, with a median response time of less than 30 min. Call volume peaked in February 2022 at 50 + calls daily, then declined as Ukraine's mental health infrastructure recovered—averaging 30 in March, 15 in April, 9 in May, and stabilizing at ∼5 per day for the rest of 2022. This decline in demand was anticipated, as Ukraine's mental health infrastructure was being rebuilt (48). Figure 2 summarizes call trends between March 1st and May 31st.

### 3.5 Standard operating procedures

Standard operating procedures followed WHO PFA guidelines, were evidence-based, and adapted to callers' needs (1, 31, 32). Below is the typical intervention structure used by the first author.

#### 3.5.1 Creating an effective empathetic connection

Began with initial text interactions, during which the therapist introduced herself and her role. The callers were given options to choose the preferred day, time, and mode of communication (via call or text) with the therapist. Focusing on developing an empathetic connection, the therapist dedicated complete attention to the callers during these initial interactions. She responded promptly and attentively, respecting and accommodating the individual preferences of each caller.

#### 3.5.2 Ensuring physical safety and a sense of security

Was crucial for many callers, especially as they faced their first wartime encounters with air-raid sirens and bombings. The therapist provided guidance on identifying safe locations nearby—places to seek shelter—and instructed them on appropriate behavior when the air-raid sirens sounded, followed by bombings. Drawing upon her experience living in Israel, where the public is well-versed in handling such situations, she shared verified practical emergency instructions with the callers.

#### 3.5.3 Initial collection of patient background information

Began with questions about the callers age, location, and who was present with them. They were then asked to identify their most pressing concerns and describe their current conditions and behaviors. Recording these responses was pivotal for identifying immediate needs and tailoring personalized interventions. Moreover, engaging the callers in this dialogue allowed them to “connect fragmented sequences” and organize their thoughts (49). This therapeutic approach demands flexibility and common sense from the therapist, who must exercise situational judgment regarding the questions asked and the amount of information gathered.

#### 3.5.4 Calming the callers and providing practical solutions to their problems

Most callers were experiencing acute stress and mental distress. Employing normalization techniques, grounding methods, and addressing immediate concerns proved highly effective in reducing distress (32). Callers were reassured that their reactions were normal, natural, and expected given the circumstances. The therapist alleviated their fear of going crazy by attributing the distress to the initiators of the war, explaining that it was they who did not act rationally and humanly. Additionally, breathing exercises and grounding techniques were taught to alleviate distress and induce calmness. Next, the therapist assisted the callers in pinpointing their primary immediate problems or concerns, discussing possible practical solutions, including specific actions.

For example, some callers sought assistance in managing feelings of anger or rage, emotions they had not experienced before the war. Following the normalization step, the therapist discussed acceptable methods for expressing anger that would be less harmful to both the callers and their loved ones. Various options were considered, including creative outlets like writing a letter to Russian President to express anger. Other callers struggled with extreme anxiety and experienced autonomic symptoms, such as shaky hands and a racing pulse, during sirens. The therapist instructed them on how to seek refuge in nearby safe spaces and taught gradual exposure techniques to desensitize to siren sounds. Each caller, following a session that included skill-building exercises, received a written summary outlining the tools and approaches discussed.

#### 3.5.5 Connecting to strengths and enhancing capabilities

Involved asking the callers about their methods of managing previous life crises, identifying their sources of support and previously used coping strategies. This component aimed to access sources of resilience that had been successful in the past. Talking about these past successes also helped to enhance callers' sense of capability, to strengthen their ability to manage their lives, and to reshape their self-image toward more positive.

#### 3.5.6 Providing information about available resources and new coping techniques

In this phase, the therapist introduced coping strategies once the caller felt calm, acknowledged their reactions as normal, and was ready to engage. The goal was to identify and implement the most suitable strategies. BASIC Ph model (31), which has been shown to be effective in Israel and other countries, was adapted to support Ukrainian citizens. The model was introduced to callers in Russian, guiding them through its various components and familiarizing them with a range of resources and coping strategies. This approach empowered callers to select strategies most relevant to their personal circumstances, thereby expanding their coping toolkit. After the session, the caller received a written summary for reference. While many callers had relied on social support and cognitive strategies before the war, they found techniques like breathing exercises and grounding methods more effective in the wartime context. These simple, practical exercises were well-suited for those confined at home. The discussion then shifted to establishing a daily routine incorporating self-care and supporting others—such as family, elderly neighbors, or wounded soldiers—fostering empowerment, control, and well-being.

#### 3.5.7 Conveying commitment to support

Unlike a typical therapy session that upholds a degree of professional neutrality, providing psychological assistance to Ukrainian civilians represents “a stance of solidarity,” showcasing the therapist's alignment and cooperation with the suffering of “the other” (50). In each session, the therapist conveyed a reassuring message: “*I’m here with you and will remain as long as you need me*.” At the session's conclusion, the therapist praised the callers' personal strength and courage, expressed confidence in the resilience of the Ukrainian nation, and offered follow-up sessions to continue support, monitor well-being, and assess the callers' utilization of new resources and coping strategies. For complex cases, efforts were made to arrange extended support with professional therapists in Ukraine or their current place of residence.

## 4 Reflective report on volunteer experience

Quantitative and empirical evidence on the impact of early PFA remains scarce. The primary challenge is that immediate support for crisis-affected populations takes priority, making structured research protocols impractical. This is especially true for informal groups like IRIS, which emerge spontaneously in response to urgent needs. Therefore, we examine the use and impact of PFA delivered through this international, telephone-based initiative qualitatively, using realist autoethnographic retrospective reflective report from an IRIS therapist.

### 4.1 Therapist's background

The first author, a trained educational psychologist fluent in Russian, specializes in psychological first aid for war victims. During the first three months of the war, she received 34 calls from Ukrainians—30 women (including patient K, described below) and four men. All callers were over 18, most under 42, with the eldest being 72. Primary concerns included acute stress, anxiety, and panic attacks. Some struggled to stay composed during sirens and explosions, feeling out of control and experiencing autonomic symptoms (e.g., shaky hands, racing heartbeat). Others were in shock, unable to accept their reality, fearing for their sanity. Many felt extreme anger, sometimes directed at family, while others faced helplessness, indecision, and loneliness. Some grappled with whether to stay home or seek refuge.

Drawing on her experience working with conflict-affected populations in southern Israel, the therapist conducted 1–7 sessions per caller, focusing on distress reduction, empowerment, and effective coping strategies. Interventions ended once callers successfully applied coping techniques or were referred to local resources. The first author reflects:

“Intensive prior training and work experience in a stress center during times of security threats have allowed me to become familiar with effective actions and techniques for providing psychological first aid. I found the Core Actions of the PFA approach and the BASIC Ph model to be the optimal and most efficient approaches for working with individuals affected by stress.”

### 4.2 Narrative case study

A woman in her fifties, we call her K, contacted the initiative at the end of May 2022. At the time, she stayed in Kiev with relatives. Originally, she was from Mariupol, a city that was under siege by the Russian Army and that suffered heavy bombing attacks for about three months. In her digital intake questionnaire, K wrote that she does not know how she can go on living. She shared that she suffered emotion distress and difficulties coping with painful memories. She functioned daily (taking care of her sister's baby).

The therapist held four phone sessions with K. During the first session, after introducing herself, the therapist asked K to share her story and discuss her concerns. K detailed her life in the besieged city. Before the war, she held a senior management position, lived in a high-rise apartment building, and served as the condo association president. As the war started, her city faced heavy bombardment, leaving residents without basic utilities like electricity and water, leading to cold and starvation. K spoke passionately about her deep sense of responsibility towards her neighbors. She recalled how, with the help of the Ukrainian military, she arranged for a huge tanker truck to deliver water to the residents. Then, she organized delivery of wooden planks to the condo residents, allowing them to build fire for warmth and boiling water. She also recalled a story of a young boy who lived in her condo and who was wounded in a bombing attack. The young woman, a professional nurse, fainted after she saw the boy's injuries and could not treat him. So, K was the only one left to help the boy. Despite the city's intense bombardments and numerous casualties, K remarked, “No one in my condo lost their lives; we all survived.” The therapist listened attentively, acknowledged the difficulty of K's experiences, and then reviewed the extraordinary events. She told K that her story revealed K's many triumphs. K expressed surprise. So, the therapist elaborated K's achievements, that included not losing her wits while treating the wounded boy and effectively caring for the condo's residents. While the therapist's reframing of K's story did not change any of the facts, it enabled K to see her experiences from a more positive perspective.

The first author reflects:

“Establishing positive short-term therapeutic connections quickly during telephonic intervention required therapists to demonstrate attentive listening, empathetic expression, and the ability to create a sense of solidarity with the callers. Providing psychological assistance to Ukrainian citizens fundamentally represented ‘taking a stand of solidarity,’ reflected in the therapist's emotional attunement and shared connection with’ the suffering of the other’ (50). During each session, the caller was reassured with the following statement: “I’m with you and will stay with you as long as you need me. We support Ukraine.”

The second session focused on K's emotional well-being. K described distressing memories that caused her pain, and anxiety. To alleviate her distress, the therapist utilized emotional normalization techniques. Emphasizing that such thoughts and feelings were common reactions to extraordinary circumstances, the therapist guided K in using her body as an anchor to manage distress. During the session, they practiced various techniques together. This involved breathing and grounding exercises, identifying the bodily sensations, and self-hugging. Additionally, they explored a self-distraction technique for releasing painful memories, reciting verses from a beloved song, and using calming personal affirmations, such as: “Now I’m in a safer place!' The therapist also guided K in recognizing automatic negative thoughts and memories, suggesting a deliberate choice not to dwell on them all day. Instead, the therapist introduced the concept of allocating a specific, limited time slot—no more than 30 min—dedicated to addressing these thoughts. This technique, known as “scheduling time to worry,” enhances one's ability to manage automatic negative thoughts, providing relief from persistent preoccupation throughout the day (36).

The first author reflects:

“The callers' culture expected them to restrain emotional expressions of anger, rage, fear, and pain, viewing such emotions as negative or undesirable. Initially, I focused on normalizing these emotions in order to acknowledge and accept them, fostering a sense of safety. Only after the caller felt secure did I introduce strategies for channeling negative emotions in a constructive manner.”

During the third session, the therapist and K explored further methods of handling distressing thoughts and memories. They discussed various approaches, including sharing her thoughts with family members, or addressing them during therapy sessions. The therapist introduced the BASIC Ph model and discussed its relevance to K's experiences. They also focused on identifying enjoyable daily activities for K, such as cooking, baking, or knitting items for her family. The therapist encouraged K to allocate dedicated time in her daily routine for these activities.

The first author reflects:

“Since 2009, I have used the BASIC Ph model to provide Psychological First Aid (PFA) to patients experiencing security-related stress. This model has proven effective with a diverse range of patients, including children—through the use of drawings—and adults, in both face-to-face and telephone-based interventions. The model helps patients identify effective coping mechanisms, become aware of their existing strengths, expand their coping strategies, regain functioning, and feel more secure. Similarly, K. was surprised and pleased to discover effective coping methods within herself, as well as to recognize and adopt new ones, such as physiological coping strategies like relaxation breathing and grounding techniques.”

During the fourth and final session, K talked about her successful experience to discuss painful memories with her family members and sought reassurance if it was acceptable. The therapist offered encouragement, affirming that whichever method K chose was acceptable. Towards the end of this session, the therapist suggested a long-term care service in Ukraine to assist with managing traumatic distress. Towards the conclusion of the session, K expressed her belief that she could overcome her condition and eventually resume a somewhat normal life*.*

The first author reflects:

“Over the course of four sessions, I felt a warm and open connection develop between us. K spoke openly, received recommendations, examined them, and chose those that suited her. She mentioned that during these sessions, she ‘felt my hand that did not let her sink, that kept her afloat.’ I, too, felt warmth and closeness toward her, as well as an appreciation for her strengths. It was important to me that after the terrorist attack in Israel, she reached out to me with concern for my well-being and words of support. I valued and understood that she was truly okay.”

Given the circumstances, no standardized distress-assessing instruments were administered before and after the intervention. In such cases, comparing pre- and post-intervention subjective self-assessments of overall functioning, proficiency in diverse coping skills, and readiness to seek further help when needed is recommended (32). K's self-assessment progressed from “*I do not know how 1 can go on living*” to “*I believe I can overcome my challenges and will eventually return to a somewhat normal life*,” indicating the intervention's effectiveness. This impression was affirmed during a subsequent follow-up call initiated by the therapist several months later. During this call, K conveyed that she had followed the therapist's advice and had sought long term therapy. She expressed feeling more organized in her life and eagerly anticipated the arrival of spring and Ukraine's victory in the war, despite neither having occurred yet. Nevertheless, she remained confident that both would eventually come. At the end of the call, she asked the therapist to record and share her story with the world.

### 4.3 Reflection on strengths and challenges

Psychological First Aid (PFA) is widely recognized as a “do no harm” approach to providing immediate support in the aftermath of a crisis (1). The first author did not encounter any instances where the intervention caused harm to those receiving assistance. All therapists involved in the initiative were mental health professionals, with formal academic training and professional experience that prepared them to deliver PFA. Many had prior experience providing PFA in various trauma contexts, enabling them to effectively transfer their skills to the telephone-based setting.

Callers reported reduced distress, enhanced sense of safety, strengthened social connections, and raised hope. A key aspect of remote PFA delivery was assisting callers in reconnecting with people in their social circles or identifying trusted individuals with whom they felt comfortable speaking, as such conversations could be soothing. Additionally, therapists helped individuals access support centers and local resources. The initiative developed relationships with volunteers and support centers in multiple cities across Ukraine, allowing therapists to provide callers with phone numbers and addresses for assistance. After stabilizing individuals emotionally, therapists explored ways in which callers could assist vulnerable community members, such as elderly neighbors, children, or injured soldiers. Encouraging active participation in helping others reinforced self-efficacy and restored a sense of control. The first author reflects:

“Two key factors enable providing effective PFA remotely. First factor was an exceptional professional preparation for delivering PFA, based both on prior experience and on ongoing training and peer support. The second was the shared linguistic and cultural background rooted in Soviet and post-Soviet norms, which facilitated rapport between therapists—many of whom had immigrated to Israel from the Soviet Union—and Ukrainian callers.”

“Research has documented the slow evolution and preservation of cultural norms over several generations, including in immigrant communities and post-soviet states (43). For instance, Soviet and post-Soviet norms emphasize controlling negative emotional expressions. As a result, callers often found it difficult to speak openly with family members about painful emotions, fearing they might cause distress or burden their loved ones (43). However, conversations with Israeli therapists provided a different dynamic. Callers were able to disclose personal experiences early in the interaction, contributing significantly to the establishment of strong rapport. The anonymity of remote intervention appeared to foster greater openness and honesty.”

“Another expression of Soviet norms emerged during initial interactions. Even in crisis situations—sometimes under fire—callers responded with notable politeness when I introduced myself. While such courtesy is not typically characteristic of people in crisis, it reflects the deeply ingrained Soviet and post-Soviet value of showing respect to professional authorities. Additionally, Soviet norms often discourage the open expression of negative emotions such as anger or rage within families to protect loved ones (43). When callers expressed distress over experiencing intense outbursts of anger—unfamiliar to them before the war—I focused first on normalizing and validating these emotions to foster a sense of safety. Only after the caller felt secure were strategies introduced to channel anger constructively, ensuring minimal harm to themselves and others.”

However, while fluency in Russian facilitated communication, it also presented challenges. Some callers refused to engage in therapy in the language of their invaders, requiring referral to Ukrainian-speaking therapists, when available. This delayed assistance and, in some cases, increased initial mistrust.

Additional challenges arose in occupied cities, where maintaining communication was difficult. Callers often could not speak on the phone due to safety concerns, requiring therapists to rely on written communication, which complicated interventions. These individuals often needed more extensive support. The first author reflects:

“One young woman from an occupied city in southern Ukraine initially focused on concerns about her parents, grandparents, and younger siblings. Her written communication extended over seven sessions (rather than the typical three to four) before she felt comfortable discussing her own emotional state.”

### 4.4 Learning and professional growth

The outbreak of war in Ukraine was a deeply shocking and painful event for many immigrants from the former USSR, leaving them feeling paralyzed and powerless. The first author reflects:

“The news of the war’s onset in Ukraine was a heavy and painful shock for me. I have lived in Israel for 30 years but was born and raised in the Soviet Union. It felt as though I were under bombardment alongside the frightened and suffering people. Photographs of tanks on snow-covered fields and the familiar names of cities where air raid sirens sounded stirred up family memories of the Second World War, intensifying the pain.”

“Interestingly, in this situation, I experienced and observed a shift in my personal and professional identity—from a psychological Israeli identity to that of an immigrant from the former Soviet Union. I felt a strong sense of obligation to assist those affected. With extensive experience and professional training in providing psychological first aid during wartime, I realized I could not remain passive—I had to do something to help the suffering people. But what could I do from afar?”

Volunteering with the initiative provided a sense of purpose and empowerment, helping many to cope with personal distress. During the first months of the initiative, daily supervision sessions played a pivotal role in therapists' success and well-being. For instance, in a session with Dr. Mendelson, therapists reflected on how the war evoked memories of World War II. They discussed the concept of “intergenerational transmission of trauma” and how volunteering in crisis intervention helped them process both personal distress and their families' historical memories of war.

Additionally, the combination of intensive training, supervision, and hands-on work with war-affected individuals significantly accelerated professional growth, particularly during the early months of the war.

### 4.5 Areas for improvement

The initiative never formally advertised its services. Therapists reached out to people in need largely via Facebook. Many callers learned about the initiative through their friends who already received assistance. Investing time and effort in promoting the visibility of the initiative could have helped reaching out to more people. Further training opportunities could enhance therapists' skills, particularly in navigating linguistic barriers and managing complex cases remotely. Expanding language accessibility and ensuring faster matching of callers to appropriate therapists could reduce delays in crisis response.

### 4.6 Limitations

This study has several important limitations. First, due to the exigencies of wartime, findings rely on subjective unstructured self-reports and observational data, limiting the ability to conduct a formal efficacy evaluation. Additionally, selection bias may be present, as only individuals who actively sought help were included, potentially excluding those in greatest need who lacked access or willingness to engage. Furthermore, volunteer bias may affect the findings—the IRIS initiative was volunteer-driven, which may limit the generalizability of results to formal crisis intervention programs. Finally, the shared linguistic and cultural background between therapists and callers likely facilitated rapport, meaning these findings may not extend to populations without similar common ground.

## 5 Conclusion

The implementation of Psychological First Aid (PFA) within an international, volunteer-based initiative utilizing digital platforms demonstrates the feasibility of delivering effective mental health support beyond traditional healthcare infrastructures. IRIS has continued operating for 3 years, demonstrating sustainability of the adopted protocol. But the critical role of international telephone-based groups during times of crises, including wars, is illustrated by the extremely high volume of calls, received during the first days of the escalated conflict—more than 50 a day. Recall that these calls were made to complete strangers, not associated with any formally accredited mental health group, living in a different country thousands of miles away, and advertising themselves on Facebook that is commonly perceived as a source of misinformation (51). The IRIS experience underscores the urgent need to develop social services “without address” (5) that transcend geographic limitations and provide crisis intervention in settings where local formal care is inaccessible.

Several studies have examined the implementation of Ruzek et al.'s Core Actions in telehealth settings, demonstrating that this PFA model can be effectively delivered through digital means (52, 53). Of these, Ching et al. (54) most closely align with the present study, describing a pilot remote mental health support program in which an international group of seven volunteers from England provided assistance to six young adults affected by the 2020 ammonium nitrate explosion in Beirut, Lebanon. While Ching et al. found the intervention largely successful, they noted that its capacity for outreach and delivery was limited due to its pilot nature and reliance on volunteers (p. 3). In contrast, the IRIS experience demonstrates that an informal international volunteer group can be scaled up to reach thousands in need and sustain operations for three years.

Informal international mental health groups can play a crucial role in bridging gaps within overburdened healthcare systems, particularly during large-scale crises. However, this also presents regulatory challenges, highlighting the need for regulatory frameworks that balance urgency and accessibility with ethical considerations and professional oversight. Critical evaluation of practices adopted by these informal organizations is essential for understanding their effectiveness, improving future implementation, and co-creating best practices for formal crisis intervention and support social services that embrace “Care Without Address” models.

By situating our study within the broader context of co-creating future social services, we highlight the importance of balancing top-down service structures with community engagement in addressing both local and global challenges. Co-creation methodologies enable practitioners, service users, and policy-makers to collectively envision and design offerings that not only respond to emerging community needs but also tap into the expertise and experiences of diverse groups—such as immigrant communities—who can contribute invaluable insights for improving well-being in their countries of origin. The web serves as a crucial connecting network between cultures, bridging geographical distances and empowering communities to share knowledge, collaborate effectively, and bolster mutual support (55). Marginalized groups can benefit significantly from these interconnections, as digital platforms and cross-border collaborations offer new avenues for resource-sharing, advocacy, and mutual support that might otherwise be difficult to access (56). This empowerment process benefits both the host country and home communities, illustrating how knowledge transfer and collaboration can span boundaries. Moreover, the collaborative and participatory nature of co-creation fosters mutual learning, ensures diverse perspectives are integrated, and helps develop more inclusive and sustainable service models. Ultimately, by grounding our work in these principles, we contribute to the evolution of forward-thinking service ecosystems that remain agile and responsive to rapidly changing realities at local, national, and global levels.

Future research should focus on standardized assessments, explore diverse cultural contexts, and examine the long-term sustainability and efficacy of informal telehealth PFA initiatives in crisis settings.

## Data Availability

The original contributions presented in the study are included in the article/Supplementary Material, further inquiries can be directed to the corresponding author.
